# The Effect of Hyperlipidemia on the Course of Diabetic Retinopathy—Literature Review

**DOI:** 10.3390/jcm11102761

**Published:** 2022-05-13

**Authors:** Anna Bryl, Małgorzata Mrugacz, Mariusz Falkowski, Katarzyna Zorena

**Affiliations:** 1 Department of Ophthalmology and Eye Rehabilitation, Medical University of Bialystok, Waszyngtona 17, 15-274 Bialystok, Poland; malgorzata.mrugacz@umb.edu.pl; 2 PhD Studies, Medical University of Bialystok, 15-089 Bialystok, Poland; mariusz.falkowski@adres.pl; 3 Department of Immunobiology and Environmental Microbiology, Medical University of Gdansk, 80-211 Gdansk, Poland; katarzyna.zorena@gumed.edu.pl

**Keywords:** diabetic retinopathy, hyperlipidemia, pharmacotherapy

## Abstract

Diabetes mellitus is a very important social issue, and its retinal complications continue to be one of the major causes of blindness worldwide. The effect of glucose level on the development of retinal retinopathy has been the subject of numerous studies and is well understood. Hypertension and hyperlipidemia have been known to be important risk factors in the development of diabetes complications. However, the mechanisms of this effect have not been fully explained and raise a good deal of controversy. The latest research results suggest that some lipoproteins are closely correlated with the incidence of diabetic retinopathy and that by exerting an impact on their level the disease course can be modulated. Moreover, pharmacotherapy which reduces the level of lipids, particularly by means of statins and fibrate, has been shown to alleviate diabetic retinopathy. Therefore, we have decided to review the latest literature on diabetic retinopathy with respect to the impact of hyperlipidemia and possible preventive measures

## 1. Introduction

Diabetes is an important social problem. According to a report issued by the International Diabetes Federation, the prevalence of diabetes mellitus (DM) in the world was estimated at 537 million in the age range of 20–79 years in 2021, with an expected increase to 783 million before 2045 [1]. The Vision Loss Expert Group announced that in 2015 diabetic retinopathy was fifth among the most common causes of preventable vision impairment globally [2], in comparison with the sixth place in their 2010 report [3].

The role of hyperglycemia in the development of diabetic lesions in the retina has been explained in detail. Hyperglycemia, hypertension and hyperlipidemia are known risk factors in the development of many vascular diseases, including diabetic retinopathy [4,5,6]. The latest results of epidemiological studies suggest that the levels of some lipoproteins are closely correlated with the incidence of diabetic retinopathy [7,8,9]. Moreover, therapies aimed at reducing lipid levels, particularly by means of statins and fibrate, have been found to alleviate diabetic retinopathy [10,11]. However, the mechanisms of this effect have not been fully elucidated.

Therefore, the aim of the current manuscript is to review the latest literature on diabetic retinopathy with respect to the impact of hyperlipidemia and possible preventive measures.

## 2. Diabetic Retinopathy

Diabetic retinopathy is a highly specific vision-threatening complication of diabetes. It develops gradually, showing progressive changes in the retinal microcirculation that lead to increased vascular permeability, retinal hypoperfusion and retinal vascular proliferation. The mechanisms of these changes have already been elucidated [12,13].

Clinically, diabetic retinopathy occurs in two major types, namely nonproliferative diabetic retinopathy (NPDR) and proliferative diabetic retinopathy (PDR). NPDR, observed as the earliest stage of diabetic retinopathy, shows severe retinal vascular permeability, the formation of microaneurysms, exudation, hard exudate and hemorrhage, leading to neurosensory retinal atrophy and ischemia and, consequently, to severe vision loss. The characteristics of the more advanced PDR include nerve fiber layer infarcts, neovascular proliferation, vitreous tortuosity and hemorrhage, leading to a higher risk of vision impairment caused by tractional retinal detachment and hemorrhage [14].

Diabetic macular edema (DME) is caused by a build-up of fluid or hard exudates in the central part of the retina—the macula. It is associated with a significant loss of vision. DME can occur at any stage of the DR.

In 2010, the incidence of DR in the DM population globally amounted to approximately 34.6% (PDR 6.96% and DME 6.81%). Thus, a large group of DM patients were found to have differently graded DR [15].

When T1DM is diagnosed, diabetic retinopathy is not present, but develops later, and after 20 years, 99% of T1DM patients have variously advanced symptoms. On the other hand, in T2DM patients, DR can be found at the time of diagnosis and twenty years afterwards and about 60% of patients have manifestations of retinopathy [16].

A growing body of evidence associates hyperlipidemia with diabetic retinopathy complications, including hard exudates and diabetic macular edema. Hard exudates are thought to be induced by the leakage of lipids from dysfunctional retinal capillaries [10]. Therefore, theses were formulated that higher levels of total cholesterol, LDL-C and TG could be considered biomarkers of the development of hard exudates in DM patients [10].

In the early stages of nonproliferative retinopathy, the patient requires strict diabetic control and, if necessary, modification of general treatment. In recent years, tremendous progress has been observed in the development of telemedicine. Many smartphone applications that have been developed allow us to obtain images of any area of the eye. This can be an effective tool in the diagnosis and monitoring of the course of diabetic retinopathy whenever contact between the patient and the doctor is difficult, like in situations caused by COVID-19 [17].

## 3. Serum Lipid Profiles and Hyperlipidemia

Cholesterol is a chemical compound belonging to the group of sterols. It occurs naturally in all living organisms. More than half of the cholesterol in the human body comes from biosynthesis (endogenous cholesterol). Its synthesis takes place mainly in the liver, less in the intestines and skin. The remaining cholesterol is supplied with food (exogenous cholesterol) [18].

Cholesterol synthesis is controlled by a feedback mechanism. Excess cellular cholesterol is converted into esters, which either accumulate in cells or combine with apolipoproteins to form VLDL fractions in the liver or intestinal chylomicrons. Cholesterol combines with proteins, phospholipids and triglycerides to form lipoproteins. Lipoproteins are made up of an inner part made of hydrophobic cholesterol esters and triglycerides, and an outer part which is made of hydrophilic proteins called apoliproteins, free cholesterol and phospholipids [18].

Lipoproteins can be divided into chylomicrons, very low density lipoproteins (VLDL-C), intermediate density lipoproteins-VLDL remnants (IDL), low density lipoproteins (LDL-C) and high density lipoproteins (HDL-C) [18]. The LDL-C has around 70–75% of total cholesterol [19].

LDL-C is metabolized in hepatocytes. Through apolipoprotein, B-100 binds to the LDL receptor domain. This complex enters the cell, where it undergoes dissociation. The LDL receptor returns to the cell surface, and LDL-C undergoes lysosomal degradation [18].

The serum lipoprotein complexes include apolipoprotein A1 (ApoA1), apolipoprotein C3 (ApoC3), apolipoprotein B (ApoB) and apolipoprotein A5 (ApoA5) [20]. The lipoproteins can be classified by taking into account their density, lipid content and the composition of various apolipoproteins. An inverse correlation was found between the ApoA1 levels and the progression of diabetic retinopathy. On the other hand, serum levels of ApoB, ApoC3 and the ApoA1 to ApoB ratio showed positive correlations with diabetic retinopathy. Serum A5 was not found to be related to the disease [21]. The ApoA1, the main structural and functional protein in the HDL molecule showing antioxidant and anti-inflammatory properties, was verified for its beneficial effect on cardiovascular and inflammatory diseases [22]. The conducted studies showed that the level of ApoA1 expression within the retinal pigment epithelium was increased in the vitreous body from diabetic patients in comparison with DM-free patients, thus showing evidence of ApoA1 protection against lipotoxicity and deposition of lipids [23].

The ApoB, being the main apolipoprotein in VLDL and LDL particles, was found to be involved in atherosclerosis and coronary diseases and correlated with the advancement of diabetic retinopathy [24].

Moreover, the circulating levels of HDL-bound apoprotein A1 and apolipoprotein B (present in LDL, lipoproteins (a), VLDL and chylomicrons) were observed to be more potent predictors of diabetic retinopathy than the traditional lipid layers [21].

As revealed by a longitudinal DCCT/EDIC cohort study conducted on unadjusted and covariate-adjusted models, the increased AGE-LDL and oxLDL levels in circulating immune complexes were related to diabetic retinopathy progression [25].

Hyperlipidemia is manifested by decreased circulating high-density lipoprotein cholesterol (HDL-C), increased circulating low-density lipoprotein cholesterol (LDL-C) and very low-density lipoprotein cholesterol (VLDL-C) [26]. Recently, evidence has revealed the role that the circulating lipid profiles play in the risk of cardio-metabolic disorders. The circulating level of LDL-C has been reported to significantly promote atherosclerosis and strongly predict atherogenesis [27].

Hyperlipidemia occurs when low-density lipoprotein (LDL), total cholesterol, triglyceride or lipoprotein levels are higher than the 90th percentile as compared to the general population or when HDL is below the 10th percentile in comparison with the general population [28].

According to this view, a few guidelines have identified LDL-C as a major target in the treatment of patients with diabetes mellitus. The serum apolipoprotein B-100 (ApoB-100) is the only apolipoprotein in the LDL particle to be recommended for the prediction of cardiovascular risk [10,29].

## 4. Studies on Lipid Abnormalities and Diabetic Retinopathy

Many studies have investigated the influence of lipid disorders on the development of diabetic retinopathy.

In the Wisconsin Epidemiologic Study of Diabetic Retinopathy (WESDR) XIII, the objective was to elucidate the association of serum cholesterol with retinopathy and the presence of hard exudates. A significant relationship was observed between the rise in cholesterol levels in insulin-dependent patients and their intensity [7].

In 1996, the ETDRS (Early Treatment Diabetic Retinopathy Study) published a report evaluating the relationship between serum lipid levels and hard retinal exudates in patients with diabetic retinopathy. It was concluded that the patients with higher serum total cholesterol or higher serum low-density lipo-protein cholesterol (LDL-C) were more likely to be diagnosed with retinal hard exudate [8]. As revealed by the United Kingdom Prospective Diabetes Study (UKPDS), higher levels of high-density lipoprotein cholesterol were correlated with more advanced retinopathy. The levels of triglycerides and low-density lipoprotein cholesterol did not seem to be associated with retinopathy severity [9].

In the ARCID study (Atherosclerosis Risk in Communities Study), retinopathy was detected in 20.5% of diabetic patients, hard exudate in 6.6%, proliferative diabetic retinopathy in 1.8% and macular edema in 1.6%. Hard retinal exudates were correlated with plasma LDL-C and lipids [30].

CHS (The Cardiovascular Health Study) was a population-based cohort study conducted to analyze and describe the relationship of retinopathy with atherosclerosis and atherosclerotic risk factors in diabetic patients. The analysis showed the relationship of retinopathy with increased mean systolic blood pressure, higher plasma total and LDL cholesterol levels and cardiovascular disease. Retinopathy was not correlated with HDL-C and plasma triglycerides [31].

The Singapore Malay Eye Study (SMES) found that increased body mass index (BMI) and higher levels of circulating LDL-C were associated with the pathogenic progression of diabetic retinopathy. These results were then repeated in another study, revealing an association between BMI and diabetic retinopathy [32]. Similarly, in a population study, called the Chennai Urban Rural Study (CURES), patients with diabetic retinopathy had higher levels of TG as compared to healthy subjects and a significant correlation was found between LDL-C and the risk of diabetic macular edema [33]. Later, the Sankara Nethralaya Diabetic Retinopathy Epidemiology and Molecular Genetic Study (SN-DREAMS) also revealed a correlation of higher levels of circulating LDL-C and increased total cholesterol/LDL-C ratio with diabetic macular edema [34].

In contrast, the MESA (The Multi-Ethnic Study of Atherosclerosis) study aimed to discuss risk factors for diabetic retinopathy in a white, black, Latino and Chinese multiethnic population in the US. The researchers failed to find a significant correlation of DR and CSME with HDL-C, LDL-C and TG in diabetic patients in the age range of 45–85 years [35].

Ucgun et al. performed a clinical study to assess the correlation between serum lipid levels and exudative diabetic maculopathy in a group of 54 patients suffering from nonproliferative diabetic retinopathy, including 27 patients with exudative diabetic macular edema (group A) and the same number of patients without this defect (group B). The serum levels of cholesterol (*p* = 0.038) and LDL-C (*p* = 0.026) showed a significant increase in group A. However, no differences were noted in the levels of TG, HDL-C and VLDL-C between the two groups [36]. Additionally, in the Sorbinil Retinopathy Trial (SRT) study, no relationship was found between hyperlipidemia and the severity of diabetic retinopathy [37].

An Italian study has highlighted a novel relationship between high HDL cholesterol and diabetic retinopathy. Sasso et al. found the HDL level of 40 mg/dL borderline. HDL levels > 60 mg/dL were associated with a high risk of DR. A relationship was observed in proliferative DR [38].

## 5. Cholesterol Metabolism and the Role of Protective Factors in the Retina

### 5.1. Cholesterol Metabolism in the Retina

Cholesterol metabolism in the retina involves the uptake from the systemic circulation, clearance and self-synthesis [39]. The blood–retina barrier (BRB) plays a key role. The retina maintains cholesterol homeostasis by strictly controlling and balancing the pathways responsible for cholesterol entry as compared to exit. The vascular endothelial cells of the retina make up the internal BRB which, when intact, does not permit cholesterol to pass. However, this mechanism becomes disturbed in conditions of hyperglycemia [39,40].

Barber et al., who used Akita mice, observed higher retinal vascular permeability after 12 weeks of hyperglycemia (at about 16 weeks of age). However, they later found morphological alterations (reduced thickness of inner plexiform and nuclear layers and a lower number of cell bodies in the ganglion cell layer), after 22 weeks of hyperglycemia, and acellular capillaries as well as morphologically changed astrocytes and microglia after 36 weeks of hyperglycemia [41].

The breakdown of the internal BRB in the retina in diabetic patients leads to the passage of lipoprotein particles to the retina, increasing the cholesterol level there. Extravasated lipoproteins have been shown to be toxic to surrounding cells. The outer BRB, built up by retinal pigment epithelial cells (RPE), allows cholesterol to be transported to the retina. Retinal cholesterol originates either from local biosynthesis or from the uptake of lipoprotein particles from the choroidal circulation by the external BRB [40,42,43]. After the uptake by RPE, cholesterol is exported by transporters ABCA1 and ABCG1 back to the choroidal circulation via reverse cholesterol transport or to the neural retina [40,43,44,45,46,47,48]. In addition to the cholesterol export, both the RPE and the neural retina metabolize cholesterol to oxysterols using the cytochrome P450 (CYP), 27A1 and 46A1 [43,44,45,46,47,48,49]. These oxysterols activate ligands for liver X receptors (LXRs). LXR activation stimulates reverse cholesterol transport genes and arrests NF-κB-mediated inflammatory gene expression. Apart from being activated by oxysterols, LXRs are controlled by the status of acetylation. Nutrient-sensing deacetylase and SIRT1 are mediated by LXR deacetylation, enhancing LXR activity. The impairment of the SIRT1-LXR axis induced by diabetes and decreased oxysterol production caused by loss of cytochromes P450, 27A1 and 46A1 in the retina lead to the reduced elimination of cholesterol, resulting in inadequate vascular repair, the activation of macrophages/microglia and widespread retinal defects [40]. The activation of LXR restores reverse cholesterol transport, prevents inflammatory conditions and the formation of acellular capillaries caused by diabetes [50]. LXR is involved not only in the regulation of lipid metabolism but is also part of the insulin signaling pathway, which takes part in the process of glucose metabolism [51].

### 5.2. Advanced Glycation End Product (AGE)

Although in healthy retina with an intact BRB, plasma lipoproteins are irrelevant, their effects can be observed after the BRB becomes deficient (like in diabetes), leading to the extravasation of lipoproteins which are then modified (i.e., oxidized and/or glycated) in tissue and become toxic towards neighboring retinal cells [40].

Glycation is a nonenzymatic process involving the reaction of sugars with amino groups, mainly proteins and lipids. The phenomenon of enhanced glycation is associated with the process of aging. In diabetes, glycation is increased due to high glucose levels and, finally, yields advanced glycation products—AGE. They bind to each other and to other proteins, disturbing the functions of cells and tissues. Additionally, they bind to cell membrane receptors, causing the formation of reactive oxygen species and activation of transcription factors. As a result, the cell is under oxidative stress and becomes damaged. High levels of AGE have been found in the vessels and nerve cells of the retina in diabetic patients [52]. The processes of increased protein glycation cause damage to the vascular endothelium and loss of connections between these cells and the inner vascular membrane, which leads to the development of diabetic microangiopathy, i.e., damage to the vascular wall, development of microaneurysms and an increase in vascular permeability [52,53]. AGEs exert many adverse effects associated with the vascular and neural complications of diabetes, such as inflammation, oxidative stress, blood clotting, fibrosis, cytotoxicity, pro- and antiangiogenic actions, disturbed cell-signaling and molecular pathways [54]. AGEs modify the circulating proteins and extravasated proteins, including LDL, which increases their pathogenicity, also in the retina [54]. In the DCCT/EDIC cohort, the baseline levels of AGE-LDL and oxidized LDL (oxLDL) in the circulating immune complexes were found to independently predict retinopathy progression a few years later [54]. The modified lipoproteins, e.g., oxidized LDL, are considered more atherogenic than the unmodified ones. They occur in higher concentrations in the vascular wall as compared to the high-turnover plasma rich in antioxidants. In diabetes, the blood retinal barriers may leak and extravasation of lipoproteins may occur. They can be remodeled when caught in the retina [54,55]. It has been proven that glycated LDL and/or oxLDL are cytotoxic towards cultured human retinal capillary endothelial cells [56], pericytes [56,57,58,59,60], retinal pigment epithelium [55]. It can also be concluded that glycated LDLs lead to changes in cell signaling, gene expression, cell apoptosis and autophagy [54,55,56,57,58,59,60,61].

Additionally, lipid β-oxidation (especially of very-long-chain fatty acids) is likely to mediate diabetes-induced rise in oxidative stress, even to a greater extent than elevated glucose catabolism, thus leading to diabetic complications [62].

### 5.3. The Role of Fibroblast Growth Factor 21 (FGF21) in Lipid Metabolism

FGF21 is a cytokine belonging to the FGF19 protein subfamily. It plays a key role in the regulation of carbohydrate and lipid metabolism. Its level is markedly elevated in the circulation in many cardio-metabolic disorders, including diabetes [63]. In T2DM patients, FGF21 reduces body weight and alleviates hyperlipidemia. In obese and diabetic mice, FGF21 was found to lower serum TG levels by regulating catabolism of lipoproteins and maintaining homeostasis of phospholipids in adipose tissue [64]. Moreover, FGF21 also enhances the utilization of lipids in reaction to the starvation of amino acids [65].

FGF21 can also modulate the δ proliferator activated nuclear receptor (PPAR-δ), and thus it is considered a key agonist of PPAR-δ, which ameliorates metabolic disorders [66]. However, FGF21 can also modulate PPAR-α and consequently influence the fat content in the body. In diabetic mice that are insulin deficient, FGF21 was found to inhibit the secretion of proinflammatory cytokines, to strengthen the antioxidant defense system of the retina and to improve retinal functioning [67].

It has also been found that FGF21 can modulate the synthesis and secretion of adiponectin, which mediates glucose and lipid metabolism in the blood [24]. Likewise, Fu et al. proved that FGF21 mediated by adiponectin can inhibit retinal neovascularization in DM mice [68].

### 5.4. Protective Effect of Retinal PPARα Activation in DR

Peroxisome proliferator-activated receptors (PPARs) and PPAR, gamma, coactivator 1 and alpha (PGC1-α) are markedly involved in the regulation of fatty acid oxidation by regulating the expression of proteins that are responsible for the uptake of fatty acids, e.g., the multifunctional fatty acid translocase CD36. In animal and human models, the regulation of PPAR signaling is highly disrupted in diabetes [69,70,71,72,73].

The CD36 is upregulated in the kidneys of diabetic animals and humans [74] and takes part in mediating the apoptotic and oxidative effects of oxidized LDL immune complexes in retinal pericytes [73,75].

The peroxisome proliferator-activated alpha receptor (PPARα) is expressed in all layers of the retina, both in T1DM and T2DM [76]. PPARα activation by fenofibrate in animal and cell models showed protective anti-inflammatory and antiapoptotic effects in endothelial cells, pericytes and RPE cells, which seem to be independent of the lipid-reducing effect. PPARα overexpression in the retina of diabetic rats markedly attenuated retinal leakage and retinitis induced by diabetes, was neuroprotective and prevented the atrophy of pericytes [71,76,77]. Moreover, the overexpression of PPARα was found to inhibit the migration and proliferation of endothelial cells [40].

## 6. Prevention

### 6.1. Diet

In order to prevent and treat chronic diseases, the consumption of total fat (<30% energy), saturated fatty acids (<10% energy) and isomers of trans unsaturated fatty acids has to be reduced. Epidemiological studies show that excessive consumption of these fats shows a positive correlation with the risk of diabetes, ischemic heart disease and cancer [78]. Saturated and trans fatty acids can accelerate atherosclerosis by increasing the concentration of total and LDL cholesterol (LDL-C) as well as through proinflammatory and prothrombotic effects.

A major source of cholesterol is derived from animal fat products, the consumption of which should be limited to 300 mg per day, and in the prevention of cardiovascular diseases to 200 mg per day [78]. The main food sources of cholesterol are egg yolks, as well as offal meats, pate and liver. Instead of animal fats, vegetable fats delivering unsaturated fatty acids should be chosen. Both n-6 monounsaturated and polyunsaturated fatty acids reduce the concentration of total and LDL-C cholesterol and elevate the concentration of HDL cholesterol. Eating fish twice a week is essential for an adequate diet. N-3 polyunsaturated fatty acids, e.g., eicosapentaenoic acid (EPA) and docosahexaenoic acid (DHA), contained in fish reduce the concentration of triglycerides [78].

Results of studies concerning the impact of fat intake on the development of diabetic retinopathy are diverse. Diabetic retinopathy was not found to be directly correlated with the consumption of fat, trans fat or total saturated fatty acid (SFA) [79,80]. Sasaki et al. found no effect of MUFA on retinopathy [79]. However, in a study conducted by Alcubierre et al. MUFA and oleic acid inversely correlated with the odds of retinopathy [80]. Some data indicate that PUFA consumption is likely to be involved in retinopathy prevention [79]. However, one study failed to find such correlations [80].

### 6.2. Physical Activity

Physical activity plays a major role in the reduction of hyperlipidemia and, in consequence, decreases the risk of diabetic complications. In T1DM patients, aerobic training increases cardiac and respiratory fitness, decreases insulin resistance and improves lipid levels and endothelial function. In T2DM, regular training reduces A1C, triglycerides, blood pressure and insulin resistance [81]. The contribution of some types of exercise, e.g., yoga and tai chi, is not well known. However, yoga may improve glycemic control, lipid levels and body composition in adults with T2DM [81]. Importantly, the beneficial effect of physical activity on the level of glycaemia should be emphasized. Physical activity is known to increase insulin resistance, thus decreasing blood glucose levels. Hyperglycemia may cause vascular damage and exceed superoxide production derived by NOX1 and NOX2. It is also involved in advanced glycation end products [82].

### 6.3. Stimulants

In the Beijing Eye Study, the consumption of alcohol was associated with a lower risk of DR in the general population [83]. Among favorable effects due to moderate alcohol intake were higher levels of high-density lipoproteins, reduced platelet aggregation and a decrease in fibrinogen level [84]. Green tea is a potent protector of diabetic retinal neurons and a regulator of the subretinal environment as it reduces ROS generation by means of the elevated expression of the glutamate transporter and restores intercellular connections and glutamine/glutamate circulation [85]. Moreover, a low dose of green tea may enhance antioxidant defense, reduce inflammatory markers and prevent the thickening of the basement retinal membrane [86].

The results of research into lipid profiles in smokers as compared to nonsmokers were analyzed. Smokers had markedly higher levels of serum cholesterol (3.0%), triglycerides (9.1%), very low-density lipoproteins (10.4%) and low-density lipoproteins (1.7%), as well as lower levels of lipoprotein cholesterol with high serum density (−5.7%) and apolipoprotein AI (−4.2%) in comparison with nonsmokers [87].

Additionally, patients who used older types of e-cigarettes had higher glycemic and triglyceride levels and lower HDL levels compared to nonsmokers. In contrast, new generation electronic cigarette users had similar lipid profiles as never-users. [88].

The consumption of coffee has been related to higher serum levels of total cholesterol and low density lipoprotein cholesterol. Two diterpenes, cafestol and kahwoel, present in coffee oil, are the main cholesterol-raising compounds in coffee; however, they are mostly removed using paper filters. Therefore, unfiltered coffee is a source of diterpenes, whereas filtered coffee does not increase serum cholesterol much [89].

Moreover, chronic stress has been found to elevate blood LDL [90].

### 6.4. Pharmacotherapy

#### 6.4.1. Statins

Both statins and fenofibrate have been shown to have beneficial effects on inhibiting diabetic retinopathy. Statins can lower total cholesterol and LDL-C, while fenofibrate is likely to reduce TG [10].

The first study, conducted in 1991 by Gordon et al., showed that the aggressive therapy of diabetic patients with hyperlipidemia may exert a positive impact on diabetic retinopathy. In patients taking pravastatin, a reduction in hard exudates and an improvement in microaneurysms were found [91].

Subsequently, the study revealed that in patients with hyperlipidemia and diabetic retinopathy who took simvastatin at a dose of 20 mg daily, an improvement in diabetic retinopathy was observed and a lower incidence of visual acuity deterioration assessed by fluorescein angiography was noted [92]. Gupta A. et al. obtained similar results. However, the researchers failed to find significant differences in the improvement of CSME regression [93].

Chung et al. performed a study involving medical records of 110 diabetic patients who were randomized to statin or placebo treatment. Statins slightly significantly inhibited diabetic retinopathy progression; macular edema was reported in 30% of patients treated with statins and in 50% in the placebo group, indicating that lipid-lowering treatment with statins inhibits the progression of diabetic macular edema [94]. A clinical trial involving over 1,648,300 diabetic patients showed that statin therapy markedly reduced the incidence of diabetic retinopathy, NPDR, PDR and vitreous hemorrhage and revealed that statin therapy is indispensable for therapy in patients with eye-threatening hyperlipidemia and hyperglycemia [95].

In 2020, Pranata et al. performed a literature review and showed that statin treatment was strongly correlated with a significantly reduced risk of developing diabetic retinopathy and reduced the need for therapeutic intravitreal injections and vitrectomy [96]. Similar results were obtained by Vail D. et al. [97].

Some studies in the available literature, however, failed to demonstrate a beneficial effect of statins on diabetic retinopathy [98]. Similar results can be found in the research called the Collaborative Atorvastatin Diabetes Study (CADS). Based on the analysis, the authors concluded that atorvastatin had the greatest role in reducing the incidence of cardiovascular diseases, but not in the prevention of diabetic retinopathy [99].

#### 6.4.2. Fenofibrate

The FIELD study focused on assessing the effectiveness of fenofibrate in cardiovascular diseases in 9795 diabetic participants. The study showed that fenofibrate reduced the frequency of laser treatment of diabetic macular edema (DME) by 31% and PDR by 30% [100].

Moreover, fenofibrate treatment was also correlated with an approximately 60% reduction in the incidence of macular edema [101].

The effect of fenofibrate was not clearly ascribed to systemic lipid-reducing effects [100], suggesting that the mechanisms may not be related to the effect of the drug on plasma lipids and/or may be related to tissue lipid processing. Therefore, fenofibrate was reported to reduce the plasma concentration of ox-LDL [102], to modulate the lectin-like ox-LDL receptor 1 (LOX-1, scavenger receptor for ox-LDL) [103] and to reduce the cellular effect of ox-LDL [104]. In animal DM models, intravitreal fenofibrate attenuated angiogenic and inflammatory responses mediated by PPARα [105].

Moreover, mechanisms not dependent on the PPARα receptor that have been demonstrated for fenofibrate [106,107] may alleviate lipotoxicity in retinal pericytes [108]. Many years ago, another fibrate drug, clofibrate [109] and, more recently, etofibrate [110], was also found to have positive effects on DR. Interestingly, while statins are generally more effective than fibrates in preventing cardiovascular problems, they appear to be less beneficial than fibrates in DR; however, they have been reported to decrease retinal hard exudates [111,112].

In the Actions of Control Cardiovascular Risk in Diabetes (ACCORD) study, a combined treatment with a statin and fenofibrate was found to reduce the advancement of diabetic retinopathy by about 40% in comparison with simvastatin alone [11,113].

#### 6.4.3. Omega-3 Fatty Acids (PUFA)

The PREDIMED (Prevención con Dieta Mediterránea) study showed that increased consumption of omega-3 long-chain polyunsaturated fatty acids (at least 500 mg/day) was associated with an almost 50% relative reduction in the risk of eye-threatening diabetic retinopathy in elderly people with T2DM [114].

Animal and cell culture studies have revealed that omega-3 polyunsaturated fatty acid (PUFA) and docosahexaenoic acid (DHA) have anti-inflammatory and antiapoptotic effects in retinal cells. A reduction in DHA due to diabetes with a concomitant rise in proinflammatory omega-6 PUFA was found to be involved in the development of DR via several mechanisms, such as omega-3 PUFA effects on plasma membrane and lipid rafts and the altered composition of oxidized fatty acid products [40].

## 7. Conclusions

The role of hyperlipidemia in the development of diabetic complications definitely requires further research. Even though the correlations between classical lipid biomarkers and diabetic retinopathy are not entirely clear, lipid-reducing therapies can be considered one of the potential therapeutic agents with a beneficial effect on the course of diabetic retinopathy. The promotion of a healthy lifestyle is extremely important among diabetic patients. Physical activity, adequate diet and avoidance of stimulants can have a positive impact on the course of diabetes and delay or alleviate its complications, including diabetic retinopathy. Patients should be made aware of the need for regular ophthalmic checkups. Early diagnosis of diabetic complications and the implementation of appropriate therapy can prevent blindness. Diabetic retinopathy does not hurt, and its initial stages may remain unnoticed by patients. It should be remembered that not only glycemic control but also a normal serum lipid profile plays a major role in the treatment of diabetic retinopathy.

## Data Availability

Not applicable.

## Abbreviations

AGE	Advanced glycation end product
ApoA1	Apolipoprotein A1
ApoA5	Apolipoprotein A5
ApoB	Apolipoprotein
ApoC3	Apolipoprotein C3
BLDL-C	Low-density lipoprotein cholesterol
BRB	The blood–retina barrier
DHA	Docosahexaenoic acid
DM	Diabetes mellitus
DME	Diabetic macular edema
EPA	Eicosapentaenoic acid
FGF21	Fibroblast growth factor 21
HDL-C	High-density lipoprotein cholesterol
Lp(a)	Lipoprotein (a)
LXRs	liver X receptors
MUFAs	Monounsaturated fatty acids
NF-Κb	Nuclear Factor-κB
NPDR	Nonproliferative diabetic retinopathy
Ox-LDL	Oxidized LDL
PDR	Proliferative diabetic retinopathy
PPARs	Peroxisome proliferator-activated receptors
PUFAs	The omega-3 polyunsaturated free acids
RPE	Retinal pigment epithelium
T1DM	Diabetes mellitus type 1
T2DM	Diabetes mellitus type 2
TG	Triglyceride
VLDL-C	Very low-density lipoprotein cholesterol
